# Study of electron spectral diffusion process under DNP conditions by ELDOR spectroscopy focusing on the ^14^N solid effect

**DOI:** 10.5194/mr-1-45-2020

**Published:** 2020-05-04

**Authors:** Marie Ramirez Cohen, Akiva Feintuch, Daniella Goldfarb, Shimon Vega

**Affiliations:** Department of Chemical and Biological Physics, Weizmann Institute of Science, Rehovot, Israel

## Abstract

Electron spectral diffusion (eSD) plays an important role in solid-state, static dynamic nuclear polarization (DNP) with polarizers that have inhomogeneously broadened EPR spectra, such as nitroxide radicals. It affects the electron spin polarization gradient within the EPR spectrum during microwave irradiation and thereby determines the effectiveness of the DNP process via the so-called indirect cross-effect (iCE) mechanism. The electron depolarization profile can be measured by electron–electron double resonance (ELDOR) experiments, and a theoretical framework for deriving eSD parameters from ELDOR spectra and employing them to calculate DNP profiles has been developed. The inclusion of electron depolarization arising from the ${}^{14}N$ solid effect (SE) has not yet been taken into account in this theoretical framework and is the subject of the present work. The ${}^{14}N$ SE depolarization was studied using W-band ELDOR of a 0.5 mM TEMPOL solution, where eSD is negligible, taking into account the hyperfine interaction of both ${}^{14}N$ and ${}^{1}H$ nuclei, the long microwave irradiation applied under DNP conditions, and electron and nuclear relaxation. The results of this analysis were then used in simulations of ELDOR spectra of 10 and 20 mM TEMPOL solutions, where eSD is significant using the eSD model and the SE contributions were added ad hoc employing the ${}^{1}H$ and ${}^{14}N$ frequencies and their combinations, as found from the analysis of the 0.5 mM sample. This approach worked well for the 20 mM solution, where a good fit for all ELDOR spectra recorded along the EPR spectrum was obtained and the inclusion of the ${}^{14}N$ SE mechanism improved the agreement with the experimental spectra. For the 10 mM solution, simulations of the ELDOR spectra recorded along the $g_{z}$ position gave a lower-quality fit than for spectra recorded in the center of the EPR spectrum. This indicates that the simple approach we used to describe the ${}^{14}N$ SE is limited when its contribution is relatively high as the anisotropy of its magnetic interactions was not considered explicitly.

## 1 Introduction

It has been recently recognized that electron spectral diffusion (eSD) plays a significant role in dynamic nuclear polarization (DNP) under static conditions (Hovav et al., 2015a; Leavesley et al., 2017). It affects the electron spin polarization gradient within the EPR spectrum as a consequence of microwave irradiation and thereby determines the effectiveness of the DNP process via the so called indirect cross-effect (iCE) mechanism (Hovav et al., 2015a). This is particularly relevant in the case of nitroxide radicals, the EPR spectra of which are in-homogeneously broadened in frozen solutions, at concentrations of 20–40 mM used in DNP applications. Hovav et al. (2015a, b), Siaw et al. (2014) and Shimon et al. (2012, 2014) observed that during constant microwave (MW) irradiation there exists an optimal radical concentration that leads to a maximum in the DNP enhancement. At this concentration the inter-electron spin dipolar interaction is sufficiently strong to generate a polarization gradient that favors an efficient iCE enhancement mechanism, while at higher concentrations the spectral diffusion saturates large parts of the EPR spectrum and spin temperature effects can be expected (Caracciolo et al., 2016; Kundu et al., 2018a, b). To monitor directly the electron depolarization during MW irradiation, Hovav et al. (2015b) measured the ELDOR signals of frozen TEMPOL solutions, under static DNP conditions, as a function of TEMPOL concentration, sample temperature and MW irradiation time. Furthermore, they developed a model (called the eSD model) that describes the depolarization process. This model is based on rate equations for the electron polarizations along the EPR spectrum, taking into account an exchange process between polarizations, in addition to the saturation effects of the MW irradiation and the spin-lattice relaxation. This eSD model introduces a fitting parameter $\Lambda^{\mathrm{eSD}}$ that defines the strength of the polarization exchange rate leading to the spectral diffusion within the EPR spectrum. Using this eSD model, experimental ELDOR spectra could be satisfactorily simulated and thus provide a feasible description of the eSD process. Subsequently, it was demonstrated that once the polarization gradient within the EPR spectrum has been determined via the eSD model simulations, the lineshape of the associated DNP spectrum could be reproduced taking into account the polarization differences between all electron pairs satisfying the cross effect (CE) condition (Hovav et al., 2015a). This approach was also implemented by Leavesley et al. (2017) when they explored the eSD process and its influence on the DNP efficiency at a magnetic field of 7 T. They also considered the effects of variations in the radical concentration, temperature and MW power on the ${}^{1}H$ -DNP spectra. Furthermore, Kundu et al. (2018b) used the eSD model to quantify the dependence of the electron polarization exchange parameter $\Lambda^{\mathrm{eSD}}$ on radical concentration and temperature.

To justify the rather phenomenological eSD model, Kundu et al. (2018a, b) performed quantum-mechanics-based calculations of the spin evolution and associated EPR spectra of the electron spins in dipolarly coupled small spin systems under DNP conditions. In the case of weak dipolar coupling constants and after adding cross-relaxation (Hwang and Hill, 1967; Kessenikh et al., 1964) to the ELDOR calculations, the results were similar to those obtained using the eSD model. In the case of strong dipolar couplings a thermal mixing mechanism in the rotating frame could provide the calculated EPR spectra under MW irradiation (Abragam, 1961; de Boer, 1976; Borghini, 1968; Goldman, 1970; Provotorov, 1962; Wenckebach, 2016; Wollan, 1976). These studies also contributed to the validity of the iCE model in the weak and the strong dipolar coupling regime.

In addition to the CE mechanism, leading to the main nuclear signal enhancements at relatively high radical concentrations, the solid effect (SE) process also influences these enhancements. This process contributes to the signal enhancements, but in addition causes some electron depolarization that in turn can influence the CE enhancement process (Hovav et al., 2015b; Leavesley et al., 2018). When nitroxide radicals are used as DNP polarizers, these SE depolarization effects arise from ${}^{1}H$ and ${}^{14}N$ nuclei hyperfine interactions (Kundu et al., 2018b; Leavesley et al., 2017). The SE-induced electron polarization depletions are highly evident in ELDOR spectra at concentrations that are below the usual concentration used for DNP, but their influence is observed also at concentrations around 20 mM, which are relevant for DNP (Harris et al., 2011; Thankamony et al., 2017). As the $\Lambda^{\mathrm{eSD}}$ constant is determined from ELDOR lineshapes, the SE effects should be taken into account in the eSD model to ensure the extraction of the correct value. The purpose of this study is to account explicitly for the effects of the SE mechanism on ELDOR lineshapes for nitroxides and to explore its influence on the extraction of $\Lambda^{\mathrm{eSD}}$ at concentrations relevant for static DNP.

We started this study by measuring ELDOR spectra of a 0.5 mM TEMPOL in DMSO frozen solution, in which the SE is the sole mechanism of depolarization, as the spectral diffusion mechanism is negligible. To analyze these ELDOR spectra we established a theoretical framework that accounts for all ${}^{14}N$ -SE and ${}^{1}H$ -SE depletions observed in these spectra. For this low concentration, the ELDOR spectrum is identical to the ELDOR-detected NMR (EDNMR) spectrum of nitroxide, which has already been studied and simulated in the past (Cox et al., 2017; Florent et al., 2011; Jeschke and Spiess, 1998; Kaminker et al., 2014; Nalepa et al., 2014, 2018). Yet, there is one major difference: under EDNMR conditions, where resolution is of prime interest, the MW irradiation period is short, in the microsecond range, and therefore relaxation processes play a limited role during that irradiation. However, under DNP conditions the duration of the irradiation is in the range of milliseconds or longer and the electron and nuclear relaxation processes influence the magnitude of the depolarization. A second, more technical, difference is that in a full field-frequency two-dimensional (2D) EDNMR spectrum the EPR dimension is usually obtained by stepping the magnetic field (Florent et al., 2011; Jeschke and Spiess, 1998; Kaminker et al., 2014; Nalepa et al., 2014, 2018) unless chirped pulses are being used (Wili and Jeschke, 2018), while 2D ELDOR maps in the context of DNP are obtained by stepping the frequency. In some earlier works the contributions from different nuclei in the EDNMR spectra were taken into account by superimposing their individual spectra, ignoring the contributions of combination frequencies (Tan et al., 2019; Wang et al., 2018). In others, the combinations were also taken into account and reproduced in the simulated spectra (Cox et al., 2017). The appearance of these lines depends on the experimental conditions (Cox et al., 2017). As under DNP conditions the duration of the microwave irradiation is long we also took into account the ${}^{14}N$ – ${}^{1}H$ combination lines in the ELDOR spectral simulations.

After analyzing the 0.5 mM spectrum, we proceeded to 10 and 20 mM TEMPOL solutions, where spectral diffusion becomes significant. We measured their ELDOR spectra and analyzed them employing the eSD model (Hovav et al., 2015b), taking into account the SE mechanism through an ad hoc inclusion of the ${}^{14}N$ and ${}^{1}H$ frequencies.

## 2 Methods and materials

### 2.1 Sample preparation

Samples of 2–3 µ L in 0.6 mm ID × 0.84 mm quartz tubes, with 0.5, 10 and 20 mM TEMPOL dissolved in a solution of DMSO / $H_{2}O$ ( 1:1 v/v), were degassed by a *freeze–pump–thaw* procedure and fast-frozen with liquid nitrogen. TEMPOL and DMSO were both purchased from Sigma Aldrich and used as is.

### 2.2 Spectroscopic measurements

All measurements were carried out on our W-band (95 GHz, 3.4 T) in-house-built EPR spectrometer (Goldfarb et al., 2008; Mentink-Vigier et al., 2013) at 20 K.

Echo-detected EPR (ED-EPR) spectra were measured using the pulse sequence π/2 - τ - π - τ -echo with τ=600 ns, while increasing the magnetic field stepwise from 3370 to 3395 mT, with a 2 ms repetition time. The pulse lengths were 100 ns for the π/2 pulse and 200 ns for the π pulse, optimized at a detection frequency of 94.90 GHz.

Electron spin-lattice relaxation times $T_{1e}$ were measured at different positions within the EPR spectrum by saturation recovery experiments with a long MW saturation pulse of 30 ms and echo pulses of 300 ns each, which is typical for DNP MW power. The $T_{1e}$ curves were analyzed using a superposition of two exponential functions with time constants $t_{1}$ and $t_{2}$ , with the slow (and major) component assigned to $T_{1e}$ .

**Figure 1:**
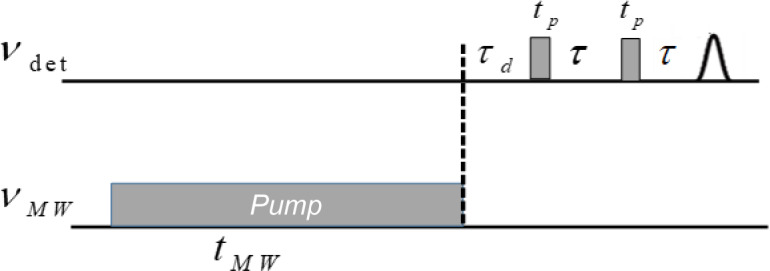
ELDOR pulse sequence, where $\nu_{\det}$ is the detection frequency, $\nu_{\mathrm{MW}}$ is the frequency of the pump pulse and $t_{\mathrm{MW}}$ is the duration of the pump pulse.

The ELDOR pulse sequence is shown in Fig. 1 and ELDOR spectra were measured at different detection frequencies along the EPR spectrum. The spectrometer was set to low power, which is typical for DNP using the detection sequence α - τ - α - τ -echo, where α is a flip angle of less than π/2 . While for EPR applications ELDOR is carried out at a fixed detection frequency and the magnetic field is varied to access different regions in the EPR spectrum, here we kept the field constant and varied the detection frequency to access the spectrum width as done for DNP applications. To carry out these ELDOR measurements, we increased the bandwidth of the cavity to accommodate the full spectrum of TEMPOL (approx. 500 MHz). The cavity resonance was tuned to 94.80 GHz. For the 0.5 mM sample ELDOR spectra (40 in total) were recorded as a function of the pump frequency, which was varied from 94.3 to 95.3 GHz. To obtain 2D ELDOR data ELDOR spectra were measured at different detection frequencies in intervals of 10 MHz from 94.55 to 94.95 GHZ, which covers most of the EPR spectrum. The amplitude of the pump pulse, $\nu_{1}$ , was 0.5 MHz, as determined by a nutation experiment at 94.8 GHz, corresponding to an inversion pulse of 1 µ s. The experimental parameters for the ELDOR experiments are listed in Table 1.

**Table 1:** Parameters used in EDNMR experiment for 0.5, 10 and 20 mM radical concentration (see Fig. 1).

$t_{p}$	T	$t_{\mathrm{MW}}$	Repetition time	$\tau_{d}$
300 ns	600 ns	10 ms	20 ms	6 µ s

## 3 Simulations

### 3.1 Low radical concentrations

### The Hamiltonian and the allowed transition

In an effort to analyze the ELDOR spectra of the 0.5 nm TEMPOL solution we rely on quantum-mechanics-based calculations considering the spin evolution of a three-spin system consisting of an electron spin, S=1/2 , coupled to a single ${}^{1}H$ nucleus and a single ${}^{14}N$ nucleus. Simulations of these ELDOR spectra were performed using a modified version of the computer code developed by Kaminker et al. (2014) for a two-spin system; one electron spin and one ${}^{14}N$ nucleus. The simulated ELDOR spectrum comprises EPR signals calculated at fixed detection frequency positions $\nu_{\det}=\omega_{\det}/2\pi$ as a function of the pump pulse frequency, $\nu_{\mathrm{MW}}=\omega_{\mathrm{MW}}/2\pi .$ In these calculations, we had to take into account the fact that the duration of the MW irradiation in DNP experiments $t_{\mathrm{MW}}$ is much longer than commonly used in EDNMR spectroscopy (millisecond vs. microsecond range, respectively). For such long irradiation times the three-spin calculations cannot account for the experimental spectral observations, mainly due to the fact that the real spin system is more extended than only three spins because of the many coupled protons present in the sample. Accordingly, without extending the number of spins in our model we had to modify Kaminker's procedure to reproduce the experimental observations, as discussed next.

The three-spin system is described by the following spin Hamiltonian in the MW rotating frame, assuming the high field approximation:

1 $$\begin{array}{rl} {\hat{H}}_{\theta ,\;\varphi } & =\Delta \omega_{e}{\hat{S}}_{z}-\omega_{N}{\hat{I}}_{zN}-\omega_{H}{\hat{I}}_{zH}+A_{zz}^{H}{\hat{S}}_{z}{\hat{I}}_{zH}+A_{zz}^{N}{\hat{S}}_{z}{\hat{I}}_{zN}\\ & +(A_{H}^{+}{\hat{I}}_{H}^{+}+A_{H}^{-}{\hat{I}}_{H}^{-}){\hat{S}}_{z}+(A_{N}^{+}{\hat{I}}_{N}^{+}+A_{N}^{-}{\hat{I}}_{N}^{-}){\hat{S}}_{z}\\ & +{\hat{I}}_{N}\cdot \tilde{Q}\cdot {\hat{I}}_{N}, \end{array}$$

where

2 $$\Delta \omega_{e}{\hat{S}}_{z}=(\mu_{B}B_{0}g_{\mathrm{eff}}(\theta ,\;\varphi )-\omega_{\mathrm{MW}}){\hat{S}}_{z}.$$

In Eq. (1) we neglected the dipolar interaction between the nuclei. $\Delta \omega_{e}$ is the off-resonance electron frequency, $B_{0}$ is the strength of the external magnetic field pointing along the z axis of the laboratory frame, and $g_{\mathrm{eff}}(\theta ,\;\varphi )$ is the effective g tensor parameter for a specific orientation of the magnetic field with respect to the principle axis system of the g tensor, given by the polar angles θ and φ . The g tensor used for the calculation is g=[2.0065,2.0037,1.9997] , obtained by simulating, using Easyspin (Stoll and Schweiger, 2006), the frequency domain EPR spectrum extracted from the echo intensity of the ELDOR spectra with the pump pulse set far outside the EPR spectrum (see Fig. S1 in the Supplement). The g values obtained from the EPR simulations and further used in the EDNMR simulations differ from those reported by Florent et al. (2011) ( g=[2.00988,2.00614,2.00194] ) as they compensate for an error of 4 mT in the determination of $B_{0}$ . These g values were used to determine the selected orientations and to calculate $g_{\mathrm{eff}}$ in Eq. (2). Because the energies and their differences depend on the product $g_{\mathrm{eff}}B_{0}$ , where the error in $B_{0}$ has been compensated in g , they are not affected by the error in the field. The shift of 4 mT in $B_{0}$ results in a shift of the proton frequency by 0.17 MHz, which is very small compared to the EDNMR linewidth. For ${}^{14}N$ it is even smaller and therefore the errors in the nuclei Larmor frequencies are negligible. The Larmor frequencies of ${}^{1}H$ and ${}^{14}N$ are $\omega_{H}=2\pi \nu_{H}$ and $\omega_{N}=2\pi \nu_{N}$ , respectively. In the EPR high field approximation the terms that contribute to the hyperfine interaction are the secular and pseudo-secular terms with coefficients $\left(A_{zz}^{H},\;A_{H}^{\pm }\right)$ for ${}^{1}H$ and $\left(A_{zz}^{N},\;A_{N}^{\pm }\right)$ for ${}^{14}N$ , where $A_{\pm }^{K}=A_{zx}^{K}\pm iA_{zy}^{K}$ , K=H,N . In the case of ${}^{14}N$ the hyperfine tensor contains an isotropic contribution $a_{\mathrm{iso}}^{N}\neq 0$ in addition to the anisotropic tensor elements $\left[a_{ZZ}^{K},\;a_{XX}^{K},\;a_{YY}^{K}\right]$ , where X , Y and Z are its principle axes. Assuming that the two anisotropic hyperfine interactions are of axial symmetry (i.e., $a_{XX}^{K}=a_{YY}^{K}=-1/2a_{ZZ}^{K})$ and that their major principal axes coincide with that of the g tensor, the hyperfine coefficients of ${\hat{H}}_{\theta ,\;\varphi }$ become $A_{zz}^{K}\equiv A_{zz}^{K}(\theta )=a_{\mathrm{iso}}^{K}+\frac{1}{2}a_{ZZ}^{K}(3{\mathrm{cos}}^{2}\theta -1)$ and $A_{\pm }^{K}\equiv A_{\pm }^{K}(\theta )=\frac{3}{2}a_{ZZ}^{K}\mathrm{cos}\theta \mathrm{sin}\theta$ (Schweiger and Jeschke, 2001). In the case of TEMPOL, the isotropic ${}^{14}N$ contribution is $a_{\mathrm{iso}}^{N}=44\;\;\mathrm{MHz}$ and the anisotropic value is $-a_{ZZ}^{N}=55\;\mathrm{MHz}$ . The ${}^{1}H$ hyperfine value was taken as $a_{ZZ}^{H}=3,\mathrm{MHz}$ . Finally, the ${}^{14}N$ nuclear quadrupole interaction is also included in the spin Hamiltonian. Here we used the principal values of the quadrupole tensor obtained by Florent et al. (2011), $\left(Q_{XX},\;Q_{YY},\;Q_{ZZ})=(0.48,\;1.29,\;-1.77\right)$ MHz, and again assumed that its principal axes coincides with those of the g tensor.

The MW irradiation Hamiltonian in the rotating frame is defined as

3 $${\hat{H}}_{\mathrm{MW}}=\omega_{1}{\hat{S}}_{x}.$$

At the start of all our simulations, the Hamiltonian for each set of (θ,φ) angles is represented in matrix form, in the 12 product states of the basis sets in the laboratory frame $|\chi_{e}\rangle$ , $|\chi_{H}\rangle$ with $\chi_{e,\;H}=\alpha ,\;\beta$ and $|\chi_{N}\rangle$ with $\chi_{N}=+1,\;0,\;-1$ , and diagonalized according to

4 $${\hat{\Lambda }}_{\theta ,\;\varphi }={\hat{D}}_{\theta ,\;\varphi }^{-1}{\hat{H}}_{\theta ,\;\varphi }{\hat{D}}_{\theta ,\;\varphi }.$$

${\hat{D}}_{\theta ,\;\varphi }$ is the diagonalization matrix and ${\hat{\Lambda }}_{\theta ,\;\varphi }$ is the diagonal matrix consisting of the eigenvalues $E_{i}^{\theta ,\;\varphi }$ , in frequency units, corresponding to the 12 eigenstates $|\lambda_{i}^{\theta ,\;\varphi }\rangle$ with i=1,…,12 . The EPR transition probabilities between levels $|\lambda_{i}^{\theta ,\;\varphi }\rangle$ and $|\lambda_{j}^{\theta ,\;\varphi }\rangle$ are as follows:

5 $$P_{i,\;j}^{\theta ,\;\varphi }=\left|2\langle \lambda_{i}^{\theta ,\;\varphi }\left|{\hat{D}}_{\theta ,\;\varphi }^{-1}{\hat{S}}_{x}\;{\hat{D}}_{\theta ,\;\varphi }\right|\lambda_{j}^{\theta ,\;\varphi }\rangle \right|{}^{2}.$$

**Figure 2:**
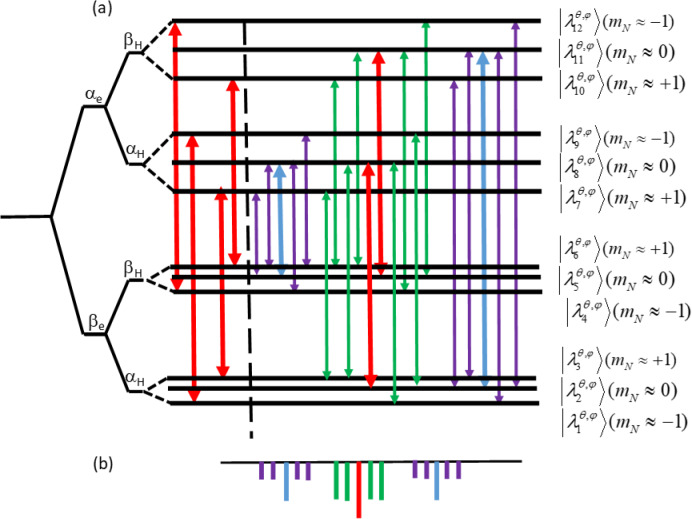
**(a)** A schematic energy level diagram of the three spin system with angles ( θ , ϕ ), corresponding to an allowed transition. The eigenstates $|\lambda_{i}^{\theta ,\;\varphi }\rangle$ are characterized by their $m_{N}$ values and product states of $|\chi_{e}\rangle$ , $|\chi_{H}\rangle$ and $|\chi_{N}\rangle$ . The energy level differences $\nu_{e}$ and $\nu_{H}\pm A_{H}$ are scaled arbitrarily. On the left of the energy level diagram the allowed transitions (3–7), (6–10), (1–9) and (4–12) are indicated by the red arrows. On the right the red arrows correspond to the allowed transition between the states with subindexes (2–8) and (5–11). The nitrogen forbidden transitions (2–9), (2–7), (4–11) and (6–11) are assigned by the green arrow and the proton forbidden transitions (2–11) and (5–8) by the blue arrows. The purple arrows indicate the combined proton–nitrogen transitions. **(b)** A schematic presentation of the ELDOR spectrum corresponding to overlapping allowed (2–8) and (5–11) transitions following the color coding of the arrows.

When $\left|Q_{ZZ}\right|<\omega_{N}<\left|\frac{1}{2}a_{ZZ}^{N}\right|,\;\left|a_{\mathrm{iso}}\right|$ , the $\omega_{N}{\hat{I}}_{z,\;N}$ term in all Hamiltonians ${\hat{H}}_{\theta ,\;\varphi }$ has little influence on the form of the eigenstates, which are products of the electron states $|\chi_{e}\rangle$ with the eigenvalues $m_{e}=\pm 1/2$ , the hyperfine mixed proton states approximately equivalent to $|\chi_{H}\rangle$ with $m_{H}\approx \pm 1/2$ and the nitrogen states $|\chi_{N}\rangle$ , mainly determined by the hyperfine interaction terms in ${\hat{H}}_{\theta ,\;\varphi }$ with $m_{N}\approx +1,\;0,\;-1$ . As a result we can easily recognize six “allowed” transitions with frequencies $\nu_{(i,\;j{)}_{a}}(\theta ,\;\varphi )=(E_{i}^{\theta ,\;\varphi }-E_{j}^{\theta ,\;\varphi })$ that correspond to EPR transitions $(i-j{)}_{a}$ , with $\Delta m_{e}=\pm 1$ , $\Delta m_{H}\approx 0$ and $\Delta m_{N}\approx 0$ and thus $P_{i,\;j}^{\theta ,\;\varphi }\approx 1$ . We note that for orientations along the x , y axis, the ${}^{14}N$ hyperfine interaction is close to $\omega_{N}$ and therefore $P_{i,\;j}^{\theta ,\;\varphi }<1$ . Figure 2 presents a schematic energy level diagram of the three-spin system for an arbitrary set of angles (θ,φ) . The six allowed transitions are indicated by red arrows. For one of these transitions the corresponding homonuclear “single quantum” (SQ) forbidden transitions, with $\Delta m_{H}\approx \pm 1$ or $\Delta m_{N}\approx \pm 1$ , are also indicated, in blue or green, respectively. The heteronuclear “double-” and “zero quantum” (DQ and ZQ) forbidden transitions, with $\Delta m_{H}\approx \pm 1$ and $\Delta m_{N}\approx \pm 1$ , are shown in purple.

Using the Orisel function in Easyspin (Stoll and Schweiger, 2006), the values of $E_{i}^{\theta ,\;\varphi }$ and $P_{i,\;j}^{\theta ,\;\varphi }$ were calculated for a collection of 9609 sets of values of (θ,φ) and from them all transition frequencies $\nu_{i,\;j}(\theta ,\;\varphi )$ were determined. To choose which orientations of the spin system contribute to the allowed EPR signal at a given $\nu_{\det}$ , we search for those sets of angles (θ,φ) for which at least one allowed transition falls in the frequency range $\nu_{\det}-3\;\mathrm{MHz}\leq \nu_{(i,\;j{)}_{a}}(\theta ,\;\varphi )\leq \nu_{\det}+3\;\mathrm{MHz}$ . This frequency span provides a frequency bandwidth of 6 MHz for the detection pulse, estimated as the excitation bandwidth for a detection pulse of 300 ns length. In addition, it can account for some g and hyperfine strains. This procedure generated a subset of selected $(\theta ,\;\varphi {)}_{\det}$ pairs for each $\nu_{\det}$ , the size of which depends on the position of $\nu_{\det}$ within the EPR spectrum.

After choosing a value for $\nu_{\det}$ we simulated the ELDOR spectra of all crystal orientations of the subset $(\theta ,\;\varphi {)}_{\det}$ . The sum of these spectra are compared with the measured ELDOR spectrum at that frequency. To obtain the individual ELDOR spectra we calculated the EPR signal at $\nu_{\det}$ after a long MW pump pulse as a function of the frequency of this pulse, $\nu_{\mathrm{MW}}$ .

### The population rate equation

To follow the evolution of the spin system during the long MW irradiation period, prior to the EPR detection, it is sufficient to consider only the eigenstate populations $p_{i}^{\theta ,\;\varphi }(t)$ of all $|\lambda_{i}^{\theta ,\;\varphi }\rangle$ for the detection subset, as described earlier (Hovav et al., 2010, 2015b). The rate equation during the MW irradiation for these populations can be presented as

6 $$\frac{d}{dt}p_{i}^{\theta ,\;\varphi }=\sum_{j=1,\;12}\left\{-R_{ij}^{\theta ,\;\varphi }+W_{ij}^{\theta ,\;\varphi }\right\}p_{j}^{\theta ,\;\varphi },$$

where $R_{ij}^{\theta ,\;\varphi }$ denotes the elements of the 12×12 spin-lattice relaxation matrix ${\hat{R}}_{\theta ,\;\varphi }$ , and $W_{ij}^{\theta ,\;\varphi }$ denotes the elements of the 12×12 MW rate matrix ${\hat{W}}_{\theta ,\;\varphi }$ . The relaxation matrix ${\hat{R}}_{\theta ,\;\varphi }$ is equal to the sum of the relaxation matrices ${\hat{r}}_{\left(ij\right)}^{\theta ,\;\varphi }$ of all transitions {i-j} with $E_{j}>E_{i}$ . The non-zero matrix elements of ${\hat{r}}_{\left(ij\right)}^{\theta ,\;\varphi }$ are derived, assuming a linear field fluctuation causing $T_{1e}$ :

7a $$\begin{array}{r} r_{(ij),\;ii}^{\theta ,\;\varphi }=-\frac{1}{T_{1,\;ij}}\frac{1}{\left(1+\eta_{ij}\right)};\;\;r_{(ij),\;ij}^{\theta ,\;\varphi }=\frac{1}{T_{1,\;ij}}\frac{\eta_{ij}}{\left(1+\eta_{ij}\right)},\\ r_{(ij),\;ji}^{\theta ,\;\varphi }=\frac{1}{T_{1,\;ij}}\frac{1}{\left(1+\eta_{ij}\right)};\;\;r_{(ij),\;jj}^{\theta ,\;\varphi }=-\frac{1}{T_{1,\;ij}}\frac{\eta_{ij}}{\left(1+\eta_{ij}\right)} \end{array},$$

and

7b $$\frac{1}{T_{1,\;ij}}=\frac{{\left|2\langle \lambda_{i}^{\theta ,\;\varphi }\left|{\hat{S}}_{x}\right|\lambda_{j}^{\theta ,\;\varphi }\rangle \right|}^{2}}{T_{1e}},$$

with $\eta_{ij}^{\theta ,\;\varphi }=p_{i}^{\theta ,\;\varphi ;\;\mathrm{eq}}/p_{j}^{\theta ,\;\varphi ;\;\mathrm{eq}}$ being the ratio between the thermal equilibrium populations defined in the laboratory frame, and

7c $${\hat{R}}_{\theta ,\;\varphi }=\sum_{\left\{i-j\right\}}{\hat{r}}_{\left(ij\right)}^{\theta ,\;\varphi }.$$

The elements of ${\hat{W}}_{\theta ,\;\varphi }$ are equal to the sum of the ${\hat{w}}_{\left(ij\right)}^{\theta ,\;\varphi }$ matrices with non-zero elements that express the effective irradiation strength on each transition (i-j) (Hovav et al., 2010):

8a $$\begin{array}{rl} & w_{(ij),\;ij}^{\theta ,\;\varphi }=w_{(ij),\;ji}^{\theta ,\;\varphi }=-w_{(ij),\;ii}^{\theta ,\;\varphi }=-w_{(ij),\;jj}^{\theta ,\;\varphi }=\\ & \frac{\omega_{1}^{2}{\left|2\langle \lambda_{i}^{\theta ,\;\varphi }\left|{\hat{S}}_{x}\right|\lambda_{j}^{\theta ,\;\varphi }\rangle \right|}^{2}T_{2\mathrm{mw}}}{1+4\pi^{2}{\left\{\nu_{ij}^{\theta ,\;\varphi }-\nu_{\mathrm{MW}}\right\}}^{2}T_{2\mathrm{mw}}^{2}} \end{array}$$

and

8b $${\hat{W}}_{ij}^{\theta ,\;\varphi }=\sum_{\left(i-j\right)}{\hat{w}}_{\left(ij\right)}^{\theta ,\;\varphi }.$$

Here $\omega_{1}$ is the MW amplitude (see Eq. 3). A transverse relaxation time $T_{2\mathrm{mw}}$ , which determines the off-resonance efficiency of the irradiation, is introduced and for simplicity is assumed to be the same for all transitions. Note that $T_{2\mathrm{mw}}$ is not the measured phase memory time, $T_{M}$ . After entering the values of $T_{1e}$ , $\omega_{1}$ and an irradiation time, it is possible to solve Eq. (6) and to use the populations at the end of the irradiation to evaluate the EPR signals.

Setting the detection frequency at one of the allowed transition frequencies and irradiating with a pump frequency that matches one of its associated forbidden transitions (i.e, they share a common energy level) result in a depletion of the EPR signal. The calculations show that the depletion can be very significant for pump pulses on the order of tens of microseconds but disappears for irradiation periods of the order of tens of milliseconds. Thus using Eq. (6) works well for calculating EDNMR spectra for short pump pulses (Kaminker et al., 2014; Ramirez Cohen et al., 2017). However, for extended periods of MW irradiation, longer than $T_{1e}$ as is applied in DNP, the simulated ELDOR signals are very weak at the forbidden transition frequencies. The reason for this is that for MW irradiations longer than $T_{1e}$ , the SE spin evolution of an electron-nuclear spin pair brings the electronic polarization back to its equilibrium value. This is, however, in contrast to the experimental results where rather intense lines were observed even for long irradiation. The reason for this discrepancy is that in reality the electron spins are interacting with several equivalent coupled nuclei, which transfer their polarization to the bulk via nuclear spin diffusion. This is particularly true when many protons are present. Accordingly, reproducing the experimental results, while still employing our simplified three-spin system model, requires modification of the simulation procedure as described next.

**Figure 3:**
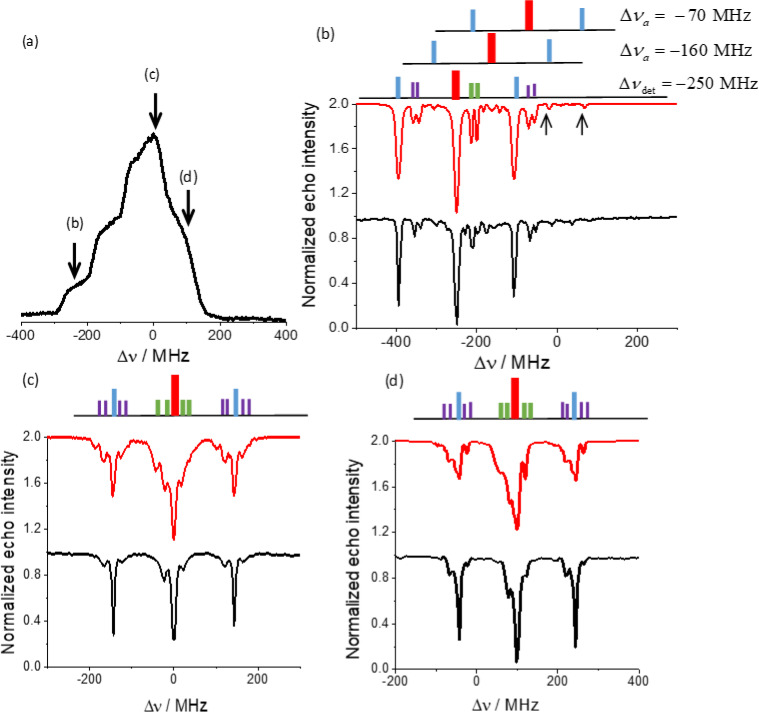
**(a)** The EPR spectrum and the positions at which the ELDOR spectra shown in **(b–d)** were recorded. **(b–d)** Experimental (black) and simulated (red) ELDOR spectra along with the associated stick spectrum using the color codes shown in Fig. 2, with detection frequencies $\nu_{\det}=94.55$ , 94.8 and 94.9 GHz, for **(b)**, **(c)** and **(d)** respectively. The frequency axis is plotted relative to the center of the EPR spectrum at 94.8 GHz such that $\Delta \nu =\nu_{\mathrm{MW}}-94.8$ GHz. The **(b)** spectrum is the most resolved; it shows the ${}^{14}N$ DQ transitions as well as peaks due to the other four allowed transitions and their associated ${}^{1}H$ forbidden transitions (indicated by arrows) arising from off-resonance and relaxation effects. A schematic for the different transitions in this case are described by the stick diagram, with $\Delta \nu_{a}$ the positions of the two pairs of allowed transitions. Experiments were performed at 20 K.

### Modification of the rate equation

In order to obtain from a three-spin calculation the observed EPR signal depletions even after long irradiation periods, we modified the form of the MW rate matrix. Realizing that an irradiation of one of the forbidden transitions, $(i-k{)}_{f}$ or $(k-j{)}_{f}$ , causes a depletion of the population difference of an allowed transition, $(i-j{)}_{a}$ , we removed the four matrix elements of ${\hat{w}}_{(ik{)}_{f}}^{\theta ,\;\varphi }$ and ${\hat{w}}_{(kj{)}_{f}}^{\theta ,\;\varphi }$ from the ${\hat{W}}_{\theta ,\;\varphi }$ matrix. This is equivalent to removing the irradiation on the forbidden transitions, which in turn cause the change in population difference of the allowed transition, $P_{i,j}^{\theta ,\;\varphi }$ . To re-introduce the effect of the forbidden transitions on $P_{i,\;j}^{\theta ,\;\varphi }$ of the allowed transitions, we added them as an artificial irradiation on the allowed one by adding them to the four non-zero matrix elements of ${\hat{w}}_{(ij{)}_{a}}^{\theta ,\;\varphi }:{\left\{{\hat{w}}_{(ik{)}_{f}}^{\theta ,\;\varphi }+{\hat{w}}_{(kj{)}_{f}}^{\theta ,\;\varphi }\right\}}_{(ij{)}_{a}}$ . In this way we ensure a depletion of the population difference of $(i-j{)}_{a}$ , without the relaxation mechanism canceling it. While realizing that the depletion of polarization due to the irradiation of the forbidden transitions can be reduced by the allowed transition relaxation dictated by the value of $T_{i,\;ij}$ , we introduce SE fitting parameters to adjust their values during irradiation: one for each of the different forbidden proton( $a_{H}^{\mathrm{SE}}$ ), nitrogen ( $a_{N}^{\mathrm{SE}}$ ), combined proton–nitrogen ( $a_{\mathrm{HN}}^{\mathrm{SE}}$ ) and even DQ nitrogen ( $a_{\mathrm{DQ}-N}^{\mathrm{SE}}$ ) transitions. In this way an irradiation on $(i-k{)}_{f}$ reproduced the experimentally observed signal depletions, still taking into account the effective MW irradiation strengths, $\omega_{1}\times \langle \lambda_{i}^{\theta ,\;\varphi }|{\hat{S}}_{x}|\lambda_{k}^{\theta ,\;\varphi }\rangle$ , and its original off resonance efficiency. Performing this procedure for all forbidden transitions, the modified ${\hat{W}}_{\theta ,\;\varphi }$ matrix contains only elements corresponding to the allowed transitions $(i-j{)}_{a}$ :

9 $$\begin{array}{rl} {\hat{W}}_{\theta ,\;\varphi } & =\sum_{\begin{array}{c} 6\;\text{allowed}\\ (i-j{)}_{a} \end{array}}{\hat{W}}_{(ij{)}_{a}}^{\theta ,\;\varphi }\;\;;\\ {\hat{W}}_{(ij{)}_{a}}^{\theta ,\;\varphi } & ={\hat{w}}_{ij}^{\theta ,\;\varphi }+a_{N}^{\mathrm{SE}}\sum_{(ik{)}_{N};(kj{)}_{N}}{\left\{{\hat{w}}_{(ik{)}_{N}}^{\theta ,\;\varphi }+{\hat{w}}_{(kj{)}_{N}}^{\theta ,\;\varphi }\right\}}_{(ij{)}_{a}}\\ & +a_{\mathrm{DQ}-N}^{\mathrm{SE}}\sum_{(ik{)}_{\mathrm{DQ}-N};\;(kj{)}_{\mathrm{DQ}-N}}{\left\{{\hat{w}}_{(ik{)}_{\mathrm{DQ}-N}}^{\theta ,\;\varphi }+{\hat{w}}_{(kj{)}_{\mathrm{DQ}-N}}^{\theta ,\;\varphi }\right\}}_{(ij{)}_{a}}\\ & +a_{H}^{\mathrm{SE}}\sum_{(ik{)}_{H};(kj{)}_{H}}\left\{{\left\{{\hat{w}}_{(ik{)}_{H}}^{\theta ,\;\varphi }+{\hat{w}}_{(kj{)}_{H}}^{\theta ,\;\varphi }\right\}}_{(ij{)}_{a}}\right.\\ & \left.+a_{\mathrm{HN}}^{\mathrm{SE}}\sum_{(kl{)}_{\mathrm{HN}};\;(lk{)}_{\mathrm{HN}}}{\left\{{\hat{w}}_{(kl{)}_{\mathrm{HN}}}^{\theta ,\;\varphi }+{\hat{w}}_{(lk{)}_{\mathrm{HN}}}^{\theta ,\;\varphi }\right\}}_{(ij{)}_{a}}\right\}. \end{array}$$

Here the sums over k and l of $(ik{)}_{K},\;(kj{)}_{K},\;(kl{)}_{KK^{\prime }},\;(lk{)}_{KK^{\prime }}$ are restricted to the homonuclear and heteronuclear forbidden transitions only. After this modification it becomes possible to write for each allowed transition $(i-j{)}_{a}$ a 2×2 rate equation for the populations $p_{i}^{\theta ,\;\varphi }(i)$ and $p_{j}^{\theta ,\;\varphi }(t)$ with a rate matrix $\left(-{\hat{r}}_{(ij{)}_{a}}+{\hat{W}}_{(ij{)}_{a}}\right)$ .

The actual relaxation pathways in the spin system are influenced by all the elements of ${\hat{R}}_{\theta ,\;\varphi }$ and as a result, an irradiation on one allowed transition can have a small effect on the populations of another allowed transition (Kaminker et al., 2014). Our modification caused this effect to vanish in the simulations. To reintroduce it we added to each ${\hat{W}}_{(ij{)}_{a}}^{\theta ,\;\varphi }$ the MW rate matrices of the other transitions ${\hat{W}}_{(kl{)}_{a}}^{\theta ,\;\varphi }$ , while introducing an additional small fitting parameter $a_{a-a}$ :

10 $${\hat{W}}_{(ij{)}_{a}}^{\theta ,\;\varphi }={\hat{W}}_{(ij{)}_{a}}^{\theta ,\;\varphi }+a_{a-a}\sum_{\begin{array}{c} (kl{)}_{a}\\ k,\;l\neq i,\;j \end{array}}{\left\{{\hat{W}}_{(kl{)}_{a}}^{\theta ,\;\varphi }\right\}}_{(ij{)}_{a}}.$$

Choosing values for all fitting parameters and inserting values for $T_{1e}$ and $T_{2\mathrm{mw}}$ , the populations of the allowed transitions corresponding to $(\theta ,\varphi {)}_{\det}$ at the end of a MW pump period $t_{\mathrm{MW}}$ at frequency $\nu_{\mathrm{MW}}$ can now be obtained using Eq. (6). The EPR signal $E_{\det}(\nu_{\det},\;t_{\mathrm{MW}})$ at $\nu_{\det}$ can then be calculated by taking the Hamiltonian diagonalization into account and by solving Eq. (6) with the modified MW rate matrices for each set of angles (φ,θ) . Adding all $\left(p_{i_{a}}^{\theta ,\;\varphi }-p_{j_{a}}^{\theta ,\;\varphi })(t_{\mathrm{MW}}\right)$ values belonging to $(\theta ,\varphi {)}_{\det}$ and normalizing their sum $S_{\det}(\nu_{\mathrm{MW}},\;t_{\mathrm{MW}})$ to the sum $S_{\det}^{\mathrm{ref}}(t_{\mathrm{MW}})$ of all $\left(p_{i_{a}}^{\theta ,\;\varphi }-p_{j_{a}}^{\theta ,\;\varphi })(t_{\mathrm{MW}}\right)$ belonging to $(\theta ,\varphi {)}_{\det}$ , obtained by again solving Eq. (6) but this time for a $\nu_{\mathrm{MW}}$ value far removed from the frequency range of all allowed and forbidden transitions, gave the following:

11 $$E_{\det}(\nu_{\mathrm{MW}},\;t_{\mathrm{MW}})=S_{\det}(\nu_{\mathrm{MW}},\;t_{\mathrm{MW}})/S_{\det}^{\mathrm{ref}}(t_{\mathrm{MW}}).$$

Plotting $E_{\det}(\nu_{\mathrm{MW}},\;t_{\mathrm{MW}})$ as a function of $\nu_{\mathrm{MW}}$ , after line smoothing over 5 MHz, results in a ELDOR spectrum at $\nu_{\det}$ . (see Fig. 2).

**Figure 4:**
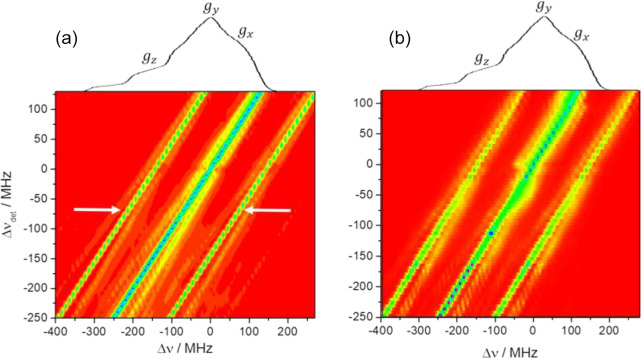
2D contour ELDOR spectra of the 0.5 mM sample **(a)** experimental (20 K) and **(b)** simulated spectra, where the y axis is the off-resonance detection frequency ( $\Delta \nu_{\det}=\nu_{\det}-94.8$ GHz) and the x axis is the off-resonance pump frequency ( Δν ). The central diagonal line corresponds to the allowed EPR transitions while the intense parallel lines on both its sides correspond to ${}^{1}H$ signals as indicated by white arrows in the experimental spectrum. The weaker lines around the center diagonal correspond to forbidden transitions involving ${}^{14}N$ and those about the outer ${}^{1}H$ lines are due to those involving both ${}^{1}H$ and ${}^{14}N$ .

### 3.2 High radical concentrations

To simulate the ELDOR spectra of the 10 and 20 mM samples we used the eSD model (Hovav et al., 2015b). This computational model divides the EPR spectrum into frequency bins and calculates the electron polarizations $P_{b}(t_{\mathrm{MW}})$ of each bin at frequency $\nu_{b}$ . It consists of a set of coupled rate equations for these polarizations with rate constants describing the effects of spin-lattice relaxation, eSD polarization exchange and MW irradiation. To take the SE into account the MW rate constants of each $P_{b}(t_{\mathrm{MW}})$ are extended by effective SE terms (Hovav et al., 2015b; Kundu et al., 2018b; Wang et al., 2018):

12 $$\begin{array}{rl} w_{\mathrm{MW}}^{b} & =\frac{\omega_{1}^{2}T_{2\mathrm{mw}}}{1+4\pi^{2}(\nu_{b}-\nu_{\mathrm{MW}}{)}^{2}T_{2\mathrm{mw}}^{2}}\\ & +\sum_{K=H,\;N,\;H-N}\frac{(A_{K}^{\mathrm{SE}}\omega_{1}{)}^{2}T_{2\mathrm{mw}}}{1+4\pi^{2}(\nu_{b}\pm \nu_{K}-\nu_{\mathrm{MW}})T_{2\mathrm{mw}}^{2}} \end{array}.$$

Here $\nu_{K}$ are the ${}^{1}H$ and ${}^{14}N$ nuclear frequencies and $A_{H}^{\mathrm{SE}}$ , $A_{N}^{\mathrm{SE}}$ and $A_{H-N}^{\mathrm{SE}}$ are fitting parameters used to scale the MW power on the forbidden transition and they just affect the SE peak intensities of the ELDOR peaks and not their positions. The eSD exchange rate constants between the polarizations in bin b and bin b ${}^{\prime }$ are defined by the exchange rate coefficients

13 $$r_{b,b^{\prime }}^{\mathrm{eSD}}=\frac{\Lambda^{\mathrm{eSD}}}{4\pi^{2}(\nu_{b}-\nu_{b^{\prime }}{)}^{2}},$$

where the parameter $\Lambda^{\mathrm{eSD}}$ determines the timescale of the spectral diffusion process. After solving the polarization rate equations for an irradiation frequency $\nu_{\mathrm{MW}}$ the polarization $P_{\det}(\nu_{\mathrm{MW}})$ at the detection frequency $\nu_{\det}$ is obtained and divided by its Boltzman equilibrium value $P_{\det}^{\mathrm{eq}}$ to obtain the ELDOR signal

14 $$E(\nu_{\mathrm{MW}},\nu_{\det},\;t_{\mathrm{MW}})=\frac{P_{\det}(\nu_{\mathrm{MW}})}{P_{\det}^{\mathrm{eq}}}.$$

## 4 Results and discussion

### 4.1 ELDOR spectra of the 0.5 mM TEMPOL

Experimental ELDOR spectra of the 0.5 mM TEMPOL were obtained by recording EPR echo intensities as a function of $\nu_{\mathrm{MW}}$ for fixed $\nu_{\det}$ and $t_{\mathrm{MW}}$ values, using the experimental parameters summarized in the Sect. 2. The results $E(\nu_{\mathrm{MW}};\;\nu_{\det},\;t_{\mathrm{MW}})$ were analyzed using the procedure described in Sect. 3. From the many ELDOR spectra measured in this way, we show in Fig. 3 (black traces) only three, each one with a different detection frequency $\nu_{\det}$ within the EPR spectrum. The dips in the ELDOR spectra, also referred to as EDNMR spectra, appear at the frequencies of the allowed and forbidden transitions, dictated by the ${}^{1}H$ and ${}^{14}N$ Larmor frequencies $\nu_{H}$ and $\nu_{N}$ and their hyperfine interactions $\left(A_{zz}^{H},\;A_{H}^{\pm }\right)$ for ${}^{1}H$ and $\left(A_{zz}^{N},\;A_{N}^{\pm }\right)$ along with the quadrupole interaction for ${}^{14}N$ (Aliabadi et al., 2015; Cox et al., 2013, 2017; Kaminker et al., 2014; Nalepa et al., 2014; Ramirez Cohen et al., 2017; Rapatskiy et al., 2012). At W-band frequencies ( ∼95 GHz) the ${}^{1}H$ frequencies are around 144 MHz and the ${}^{14}N$ frequencies are in the range ∼20 –70 MHz, as reported earlier in EDNMR experiments (Florent et al., 2011; Kaminker et al., 2014; Nalepa et al., 2014; Wili and Jeschke, 2018). Thus we expect in addition to the homonuclear forbidden transition signals additional signals around -144 , 0 and +144 MHz each with a possible spread of -70 – +70 MHz, due to the heteronuclear forbidden transitions.

**Figure 5:**
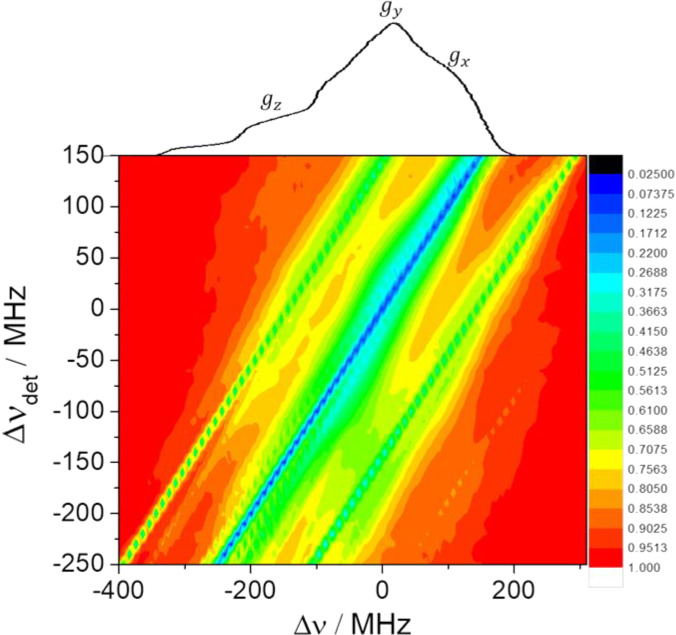
Experimental 2D ELDOR spectra of 10 mM TEMPOL solution at 20 K.

**Figure 6:**
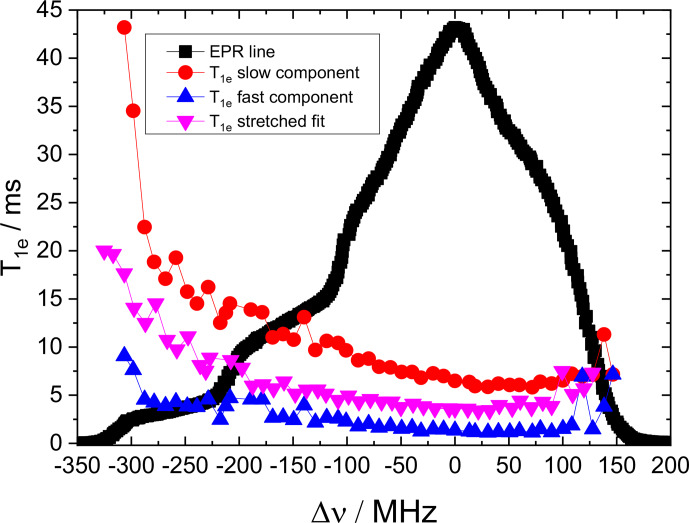
The frequency dependence of $T_{1e}$ of 10 mM TEMPOL at 20 K, measured every 10 MHz . Each point corresponds to a measurement fitted with a bi-exponential fit as noted on the figure.

Figure 3b shows the ELDOR spectrum for $\nu_{\det}=94.55$ GHz, where this frequency falls in the $g_{z}$ region of the EPR spectrum (Fig. 3a), which is characterized by its “single-crystal-like” features. As a result the ${}^{14}N$ signals are only slightly powder broadened and well resolved (Florent et al., 2011; Kaminker et al., 2014). At this detection frequency the contributions to the echo signal originate only from the two low-frequency allowed transitions (red in the $\Delta \nu_{\det}=-250$ MHz stick diagram), split by the ${}^{1}H$ hyperfine interaction, of the crystallites belonging to the “single crystal”. The MW excitation is not selective enough to resolve the protons splitting. In Table S1 in the Supplement the frequency assignments of the lines in the ELDOR spectra are correlated to the $(i-j{)}_{a}$ and $(i-j{)}_{f}$ transitions in Fig. 2, together with the color coding in the stick spectrum shown in Fig. 3b. The assignments of the other four allowed transitions are also tabulated, together with their ${}^{1}H$ and ${}^{14}N$ homonuclear forbidden transitions and the ${}^{1}H$ – ${}^{14}N$ heteronuclear forbidden transitions. In the ELDOR spectra the two ${}^{1}H$ transitions (in blue) and the four ${}^{14}N$ transitions (in green) are clearly present. The ${}^{1}H$ – ${}^{14}N$ transitions (in purple) are also detected. The additional spectral features must originate from the four non-directly detected allowed transitions with their forbidden transitions. Stick spectra of these allowed transitions and their ${}^{1}H$ forbidden transitions are also added in Fig. 3b, and it is interesting to see that some of these lines appear in the experimental ELDOR spectrum (marked by arrows in Fig. 3b). The appearance of signals corresponding to the non-directly excited allowed transition has been reported earlier (Kaminker et al., 2014) and was attributed to the combination of off-resonance and relaxation effects. In Fig. 3c the experimental ELDOR spectrum at $\nu_{\det}=94.8$ GHz $\left(g_{y}\right)$ is plotted and a schematic stick spectrum is added on the top. All possible allowed transitions contribute to this spectrum and the spectral features are broadened and even hard to distinguish. The stick spectrum represents only one typical contribution to the observed powder spectrum. The same is true for the spectrum in Fig. 3d at $\nu_{\det}=94.9$ GHz $\left(g_{x}\right)$ .

To simulate the experimental ELDOR spectra we needed to measure the $T_{1e}$ values. These were measured at several frequency positions within the EPR spectrum: 20.8 ms at $\nu_{\det}=94.6$ GHz, 13.8 ms at $\nu_{\det}=94.8\;\mathrm{GHz}$ and 15.8ms at $\nu_{\det}=94.9\;\mathrm{GHz}$ , with the highest value obtained for the $g_{z}$ region. In the simulations we used the average value of $T_{1e}=16.7\;\mathrm{ms}$ .

**Figure 7:**
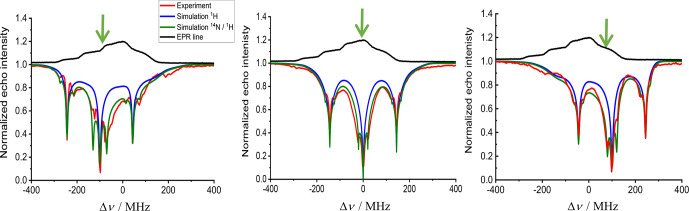
Experimental (red) and simulated (blue and green) ELDOR spectra of 10 mM TEMPOL at different positions along the EPR spectrum (in black) measured at 20 K. The green arrow indicates $\Delta \nu_{\det}$ . All spectra were fitted with $\Lambda^{\mathrm{eSD}}=60$ µ s ${}^{-3}$ , $T_{1e}=5.7$ ms and $T_{\text{2MW}}=100$ µ s. The blue spectra show the result of the simulation including only the ${}^{1}H$ while the green spectra include both ${}^{1}H$ and ${}^{14}N$ SE contributions. The detection frequency is marked with a green arrow at the top of each panel. The simulation was performed using 350 frequency bins with a 2 MHz width, spanning the whole EPR spectrum. The pump frequency spanned 1000 MHz with steps of 2 MHz; the forbidden transition fitting parameters were $A_{H}^{\mathrm{SE}}=3\times 10^{-3}$ , $A_{N}^{\mathrm{SE}}=1.5\times 10^{-3}$ and $A_{\mathrm{HN}}^{\mathrm{SE}}=0.4\times 10^{-3}$ . The NMR frequencies (corresponding the $\nu_{K}$ in Eq. 12) used in the simulation were $\upsilon_{\text{H\_NMR}}=\pm 144$ MHz, $\upsilon_{\text{N\_NMR}}=\pm 20$ MHz for ${}^{14}N$ and $\upsilon_{\text{HN\_NMR}}=\upsilon_{H}\pm 20$ MHz for the ${}^{1}H$ and ${}^{14}N$ combinations.

The best-fit simulated spectra that resemble the three experimental ELDOR spectra in Fig. 3 are shown in red. To achieve these spectra we used the following parameters: $T_{2\mathrm{mw}}=100$ µ s, $t_{\mathrm{MW}}=100\;\mathrm{ms}$ and the SE fitting parameters $a_{H}^{\mathrm{SE}}=10^{3}$ , $a_{N}^{\mathrm{SE}}=0.5$ , $a_{H-N}^{\mathrm{SE}}=10^{3}$ and $a_{a-a}^{\mathrm{SE}}=0.5\times 10^{-3}$ . These parameters were determined via manual fitting of the intensities of the different lines in the spectrum in Fig. 3b. The same parameters were used for the simulated spectra in Fig. 3c and d. The fact that the SE parameter of the ${}^{1}H$ forbidden transitions is large seems to be connected with the many protons involved in the SE process in the sample. In addition to the abovementioned forbidden transitions, we also added ${}^{14}N$ double quantum effect in the simulations by introducing a SE parameter of $a_{\mathrm{DQ}-N}^{\mathrm{SE}}=5$ . Comparing the simulated and experimental spectra we observe all expected forbidden transitions and some lines originating from the non-observed allowed transitions and their forbidden transitions. The double quantum lines expected around Δν=200 MHz are not clearly resolved. The calculated spectra in Fig. 3c and d resemble the experimental spectra, although the relative intensities of the lines do not agree so well.

A contour plot of the experimental 2D-ELDOR spectrum of the 0.5 mM sample is shown in Fig. 4a. The positions of the lines corresponding to the allowed transitions appear at the intense central diagonal of the spectrum. The signals associated with the $\left\{e-{}^{14}N\right\}$ forbidden transitions are close to the central diagonal and clearly reveal the anisotropic character of the hyperfine interaction. Namely, the strongest shifts of the line positions, with respect to the allowed line positions, are about 40 MHz in the $g_{z}$ region of the EPR spectrum and reduce to 20 MHz in the $g_{x,\;y}$ regime. The signals associated with the $\left\{e-{}^{1}H\right\}$ forbidden transitions are the intense lines parallel to the diagonal and are surrounded by the signals coming from the $\left\{e-{}^{1}H--{}^{14}N\right\}$ forbidden transitions. Figure 4b shows the simulated 2D-ELDOR contour plot, which reproduces most of the features observed in the experimental contours. Some discrepancies can be observed in the intensities of the forbidden transition lines which can be attributed to the simplifications of the model.

### 4.2 ELDOR spectra of 10 mM and 20 TEMPOL

The 2D ELDOR spectrum for a 10 mM TEMPOL solution, presented in Fig. 5, displays the main features of the ${}^{1}H$ SE solid effect lines, which run parallel to the diagonal. ${}^{14}N$ and combination lines are detectable but they are not as nicely resolved as in the 0.5 mM sample. In addition, broad features that correspond to the depolarization of the electron spins owing to the eSD process are evident. To consider both SE and eSD effects we simulated the ELDOR spectra using the eSD model, including the influence of ${}^{14}N$ and ${}^{1}H$ SE by incorporating the SE features as described in Sect. 3 and Eq. (12). We also measured $T_{1e}$ along the EPR spectrum and the results are given in Fig. 6. $T_{1e}$ displays anisotropic behavior; namely it depends on the position within the EPR spectrum with the largest variations observed in the $g_{z}$ region (similar to our earlier observation for the 0.5 mM solution). Similar $T_{1e}$ variations was also reported by Weber et al. (2017). To include the experimental $T_{1e}$ values in the simulations, we assigned to each group of five consecutive bins, each one with a width of 2 MHz, the value of $T_{1e}$ measured at the position in the EPR spectrum that correspond to those bins. Examples of experimental and simulated ELDOR spectra for three positions of the detection frequency in the EPR spectrum are shown in Fig. 7.

Initially the spectra were simulated using the eSD model considering only the ${}^{1}H$ SE effect (blue traces in Fig. 7), and the best fit gave an eSD parameter of $\Lambda^{\mathrm{eSD}}=60$ µ s ${}^{-3}$ . A better fit was obtained when taking into account ${}^{14}N$ SE, including the ${}^{14}N$ – ${}^{1}H$ combinations (green traces). This addition broadened the ELDOR lines, resulting in a better match with the experimental result, with the same $\Lambda^{\mathrm{eSD}}$ value. Nevertheless, when $\nu_{\det}$ reached the $g_{z}$ region of the EPR spectrum (Fig. 7a, $\Delta \upsilon_{\det}=-100$ MHz), the fit was not as good as in $g_{x}$ (Fig. 7b, $\Delta \upsilon_{\det}=0$ MHz) and $g_{y}$ (Fig. 7c, $\Delta \upsilon_{\det}=100$ MHz). This implies that $\Lambda^{\mathrm{eSD}}$ might be anisotropic, which is unexpected. At this point we attribute this “apparent” anisotropy to the oversimplified ad hoc inclusion of the SE mechanism into the eSD model which does not fully account for the anisotropy of the ${}^{14}N$ hyperfine interaction.

**Figure 8:**
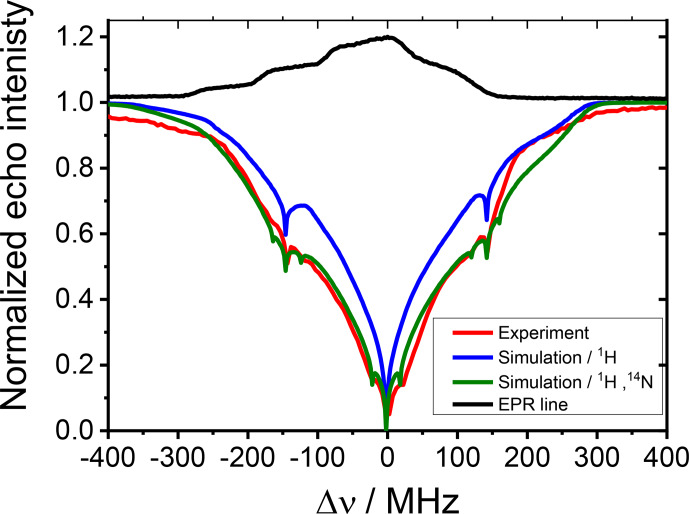
Experimental (red) and simulated (blue and green) ELDOR spectra of 20 mM TEMPOL recorded at the maximum of the EPR spectrum (shown in black). The fit was achieved with $\Lambda^{\mathrm{eSD}}=400$ µ s ${}^{-3}$ , $T_{1e}=5.7$ ms, $T_{2}=100$ µ s. The blue spectra show the result of the simulation including only the ${}^{1}H$ while the green spectra include both ${}^{1}H$ and ${}^{14}N$ SE contributions. The forbidden transition fitting parameters were $A_{H}^{\mathrm{SE}}=3\times 10^{-3}$ , $A_{N}^{\mathrm{SE}}=5\times 10^{-3}$ and $A_{\mathrm{HN}}^{\mathrm{SE}}=0.4\times 10^{-3}$ , and the nuclear frequencies were the same as in Fig. 7.

To examine the degree of the influence of the ${}^{14}N$ SE on the electron depolarization at higher radical concentrations, where the ELDOR spectrum is shaped primarily by the eSD process, we also tested the 20 mM sample and used the eSD model to simulate the ELDOR lineshape recorded with $\nu_{\det}$ set to the center of the EPR spectrum, as shown in Fig. 8. Because of the high electron spin concentration, the eSD causes a large degree of depolarization of the EPR spectrum, which translates to extensive broadening of the ELDOR spectrum.

Figure 8 shows in red the experimental ELDOR spectrum, where although the lineshape of this spectrum is determined by the eSD process, we can still see small signals coming from the ${}^{14}N$ SE. Simulation including both the ${}^{1}H$ and ${}^{14}N$ SE with $\Lambda^{\mathrm{eSD}}=400$ µ s ${}^{-3}$ gave a good agreement with the experimental spectrum. In contrast, setting $\Lambda^{\mathrm{eSD}}=400$ µ s ${}^{-3}$ and taking into account only the contributions of the ${}^{1}H$ SE did not result in a good fit. This shows that even at relatively high radical concentrations, the effect of the depolarization due to the ${}^{14}N$ SE can still be significant and if not included can introduce inaccuracies in the eSD parameters and thus also in the DNP spectra, derived from the depolarized EPR lineshapes that are constructed using these parameters. Earlier measurements showed that for a 20 mM TEMPOL concentration, ELDOR spectra measured at the $g_{y}$ and $g_{z}$ position gave the same quality fit with the same $\Lambda^{\mathrm{eSD}}$ , implying that at this concentration the relative contribution of the ${}^{14}N$ SE mechanism is small and can be accounted for by the simple model presented in this work.

## 5 Conclusions

In this work we use ELDOR measurements to determine the contributions of the ${}^{14}N$ SE to the depolarization gradient within the EPR spectrum of TEMPOL during long MW irradiation, as commonly used in DNP measurements. For a low concentration (0.5 mM) TEMPOL sample, where the SE dominates and eSD is negligible, we have successfully reproduced all the SE-related depolarization signals, including those involving combinations of ${}^{1}H$ – ${}^{14}N$ associated forbidden EPR transitions and those arising from off-resonance effects. Subsequently, we used the eSD model (Hovav et al., 2015c) to simulate ELDOR spectra of 10 and 20 mM TEMPOL samples with ad hoc addition of electron depolarization due to the ${}^{14}N$ SE based on the frequencies determined from the 0.5 mM sample. We observed that simulations including the ${}^{14}N$ SE improved the fit with experimental ELDOR spectra for the 10 mM sample. However, we noticed that at the $g_{z}$ region of the EPR spectrum the fit was not as good, indicating that the model is does not account sufficiently well for the large ${}^{14}N$ SE contributions in this region. For the 20 mM concentration the model works well and the ${}^{14}N$ SE effect is still significant and can affect the best fitted value of $\Lambda^{\mathrm{eSD}}$ . We conclude that including ${}^{14}N$ SE in the eSD model is essential for obtaining reliable fitting at high radical concentrations.

## Supplement

10.5194/mr-1-45-2020-supplement The supplement related to this article is available online at: https://doi.org/10.5194/mr-1-45-2020-supplement.

## Data Availability

The data in the figures are available at https://doi.org/10.5281/zenodo.3757682 (Ramirez-Cohen et al., 2020). The software used for simulations is available upon request.
