# Supplementary material for: Study of electron spectral diffusion process under DNP conditions by ELDOR spectroscopy focusing on the 14N solid effect
Source: Magn Reson (Gott). 2020 May 4;1(1):45–57. doi: 10.5194/mr-1-45-2020 (PMC10500736; doi:10.5194/mr-1-45-2020)
Supplement: The supplement related to this article is available online at: https://doi.org/10.5194/mr-1-45-2020-supplement. [file mr-1-45-supplement.pdf]

Supplement of Magn. Reson., 1, 45–57, 2020
https://doi.org/10.5194/mr-1-45-2020-supplement
© Author(s) 2020. This work is distributed under
the Creative Commons Attribution 4.0 License.

Open Access
MAGNETIC RESONANCE

Supplement of

Study of electron spectral diffusion process under DNP conditions by ELDOR spectroscopy focusing on the ^{14}N solid effect

Marie Ramirez Cohen et al.

Correspondence to: Daniella Goldfarb (daniella.goldfarb@weizmann.ac.il) and Shimon Vega (shimon.vega@weizmann.ac.il)

The copyright of individual parts of the supplement might differ from the CC BY 4.0 License.

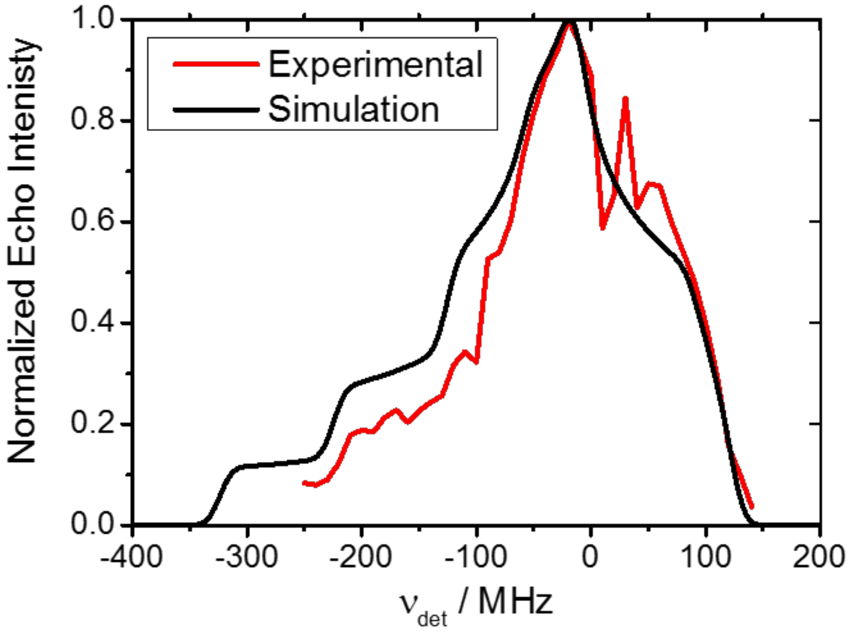

Figure S1. The frequency swept EPR spectrum (red) recorded for B=3379 mT as a function of the detection frequency, and the simulated spectrum (black).

Table S1: Assignments of the different transition frequencies (in MHz) for the orientation contributing to the measurements shown in Fig 3A. The three columns are for the three pairs of allowed transitions. In cases where the splitting of the pair is a result of the proton hyperfine the transition energy of the pair is assumed to be the same. A schematic energy level diagram is shown in Figure S2 showing the ordering of the different energy levels. The numbering of the levels is from top to bottom.

transitions	$\Delta\nu_{det} = -250$ MHz	$\Delta\nu_a = -160$ MHz	$\Delta\nu_a = -70$ MHz
Allowed (red)	(3-7);(6-10): **-251**	(2-8);(5-11): **-161**	(1-9);(4-12): **-71**
^{1}H-forbidden: (blue)	(6-7) : **-395**<br>(3-10) : **-107**	(5-8) : **-305**<br>(2-11) : **17**	(4-9) : **-215**<br>(1-12) : **72**
^{14}N-forbidden: (green)	(3-8);(6-11): **-213**<br>(2-7);(5-10): **-198**	(2-9);(5-12): **-128**<br>(2,7);(5-10): **-198**<br>(1-8);(4-11): **-104**<br>(3-8);(6-11): **-213**	(1-8);(4-11): **-104**<br>(2-9);(5-12): **-128**
^{1}H-^{14}N-forbidden: (purple)	(6-8) :**-357**<br>(5-7) : **-325**<br>(3-11) : **-69**<br>(2-10) : **-55**	(4-8) : **-248**<br>(6-8) : **-357**<br>(5-9) : **-273**<br>(5-7) : **-325**<br>(1-11) : **40**<br>(3-11) : **-69**<br>(2-10) : **-55**<br>(2-12) : **15**	(4-8) : **-248**<br>(5-9) : **-273**<br>(1-11) : **40**<br>(2-12) : **15**

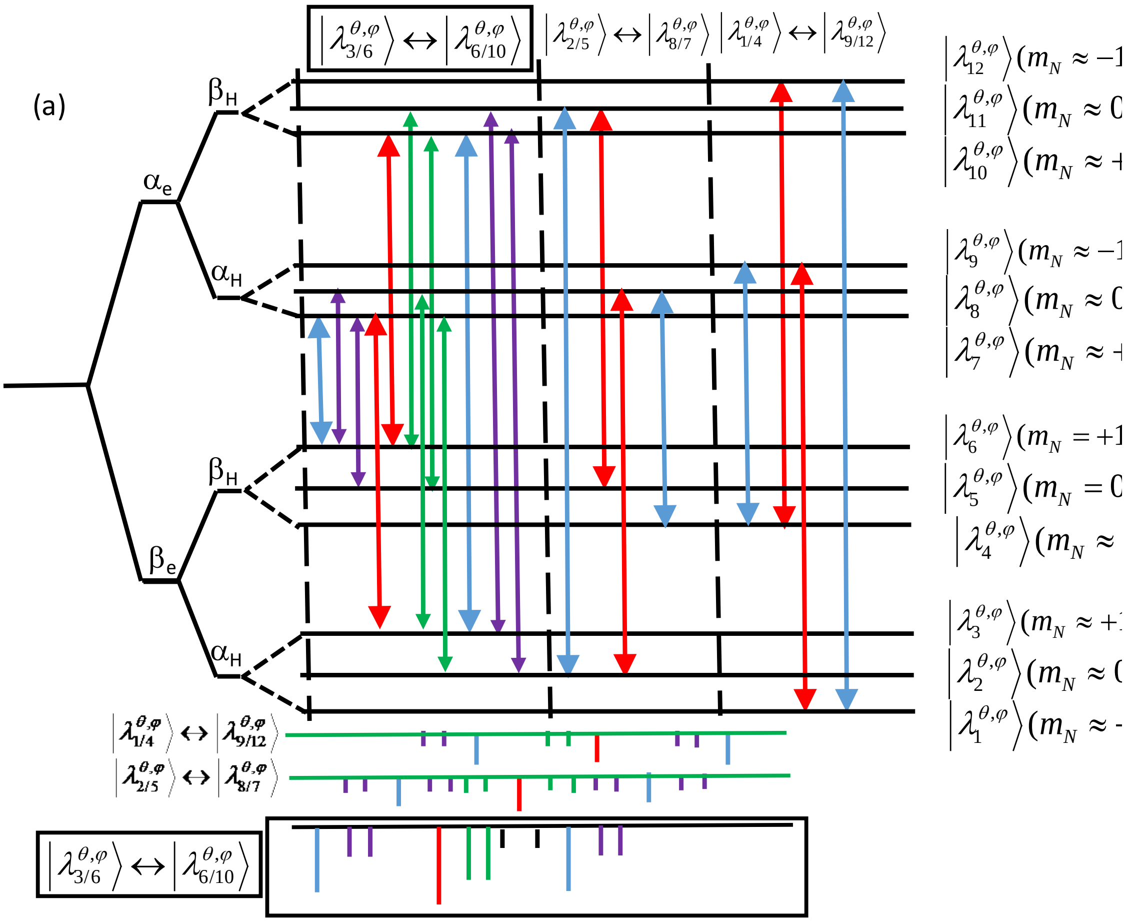

Figure S2: (a) Energy level diagram for the system described in Table S1 (b) A schematic presentation of the ELDOR spectrum following the color coding of the arrows.
